# An Automatic Detection Model for Low-Contrast Discrete Defects on Aluminum Alloy Wheels

**DOI:** 10.3390/s26010177

**Published:** 2025-12-26

**Authors:** Jian Yang, Ping Chen, Mingquan Wang

**Affiliations:** School of Information and Communication Engineering, North University of China, Taiyuan 030051, China; b20240521@st.nuc.edu.cn (J.Y.); b202505003@st.nuc.edu.cn (P.C.)

**Keywords:** defect detection, transformer, Mamba, aluminum alloy wheels

## Abstract

X-ray-based non-destructive testing technology plays a crucial role in the quality monitoring of aluminum alloy wheel hubs. Due to the characteristics of the casting process, wheel hub images often exhibit low contrast and a discrete distribution of defect edges. Existing methods often face problems such as poor feature extraction capability, low efficiency of cross-scale information fusion, and susceptibility to interference from complex backgrounds when detecting such defects. Therefore, this study proposes an innovative detection framework for defects in aluminum alloy wheel hubs. The model employs data preprocessing to enhance the quality of original images; integrates an asymmetric pinwheel-shaped convolution (PConv) with an efficient receptive field, enabling efficient focus on the edge feature information of discrete defects; innovatively constructs a Mamba-based two-stage feature pyramid network (MFDPN), which improves the network’s defect localization capability in complex scenarios via a secondary focusing-diffusion mechanism; and incorporates a channel and spatial attention block (CASAB), strengthening the model’s ability to resist interference from complex backgrounds. On our self-built wheel hub defect dataset, the proposed model outperforms the baseline by 7.2% in mAP50 and 5% in Recall at 39 FPS inference speed, thus validating its high practical utility for automated aluminum alloy wheel hub defect detection.

## 1. Introduction

As a crucial load-bearing and transmission component of automobiles, the quality of the wheel hub directly affects the safety and service life of the entire vehicle. In recent years, with the continuous improvement of requirements for automotive lightweighting and energy conservation and emission reduction, aluminum alloy—with its low density, excellent thermal and electrical conductivity, and good plasticity—has become the mainstream raw material for industrial wheel hub production [1]. The manufacturing processes of aluminum alloy wheels include low-pressure casting, gravity casting, forging and spinning forming, etc. Low-pressure casting has higher production efficiency, better dimensional consistency and lower defect rate of finished products compared with other methods, and has become one of the main processes for manufacturing aluminum alloy wheels [2]. However, due to the complexity and inherent characteristics of the casting process itself, aluminum alloy wheels are highly prone to defects during the die-casting process [3]. These defects not only reduce the mechanical properties of the wheels but may also cause safety hazards during use [4]. Early detection of substandard products can effectively prevent safety accidents and reduce maintenance costs for enterprises in the later stage.

Non-destructive testing (NDT) refers to the inspection methods that identify surface and internal defects of materials or components without damaging the inspected object or affecting its subsequent performance. At present, commonly used non-destructive testing methods include ultrasonic testing [5], infrared thermal imaging [6], radiographic testing [7], and visual inspection. Compared with other ray source detection methods, X-ray detection technology can present the density distribution and structural defects inside metals with high resolution. It has advantages such as strong traceability and wide adaptability, and has been widely applied in industrial inspection practices [8,9,10]. However, in the quality inspection processes of most wheel manufacturers today, the traditional method of manual visual inspection is still widely used. Operators rely on their own experience to manually interpret X-ray imaging results to determine whether there are defects inside the wheel. This process has many drawbacks, such as high labor costs, personal subjective biases, and inability to work efficiently for long periods of time.

In recent years, with the development of deep learning, AI-based aluminum alloy defect detection methods, which feature high precision and efficiency, are gradually replacing manual detection methods [11,12]. Existing defect detection methods mainly employ deep convolutional neural networks (CNNs) or Transformer architectures for defect localization and classification. For instance, ref. [13] introduced a reparameterized convolution module based on over-parameterization into YOLOv5, achieving a coordinated improvement in accuracy and speed for defect detection tasks. Du et al. [14] proposed a Soft-IOU optimization evaluation criterion considering the blurred boundary characteristics of defects, taking into account complex defect scenarios where a single ground truth box contains multiple predictions and vice versa. Mery D [15] suggested training YOLOv5 with simulated elliptical defects to address the overfitting issue caused by repeatedly using the same defect perspectives in traditional models. Wu et al. [16] proposed adaptive noise reduction of X-ray images to improve image quality, thereby improving the model’s ability to detect wheel hub defects. Although CNN-based defect detection models have achieved remarkable results, their limited receptive fields make it difficult to fully capture the edge contour information of blurred defects. The Transformer, with its self-attention mechanism, is particularly suitable for visual tasks requiring long-range dependency modeling and is thus increasingly popular in industrial defect detection. Li et al. [17] applied Prconv to aluminum alloy casting defect detection based on DETR, reducing computational complexity while enhancing spatial feature extraction capabilities. Ye et al. [18] proposed focusing on defect regions through a deformable attention mechanism to address issues such as large-scale differences in surface defects and complex background textures of aluminum alloys, significantly improving the ability to capture key information. However, the methods mentioned above do not fully consider the characteristics of aluminum alloy wheel defect images under X-ray: the contrast between defects and the background is low, and the defect shapes are random and mostly distributed discretely. This leads to weak defect localization capabilities of the model and an inability to accurately and efficiently identify defect edge information. Therefore, under the requirement of real-time performance for enterprises, it is particularly important to study a defect detection model for aluminum alloy wheel hubs that is suitable for such complex situations.

Recently, DEIM, as a new generation end-to-end detection framework, has demonstrated outstanding performance in the task of aluminum alloy defect detection [19]. This framework adopts a dense one-to-one (Dense O2O) matching strategy and a Matchability-Aware Loss (MAL) function, not only achieving a supervision density similar to One-to-Many (O2M), but also significantly accelerating the convergence speed of DETR [20] by imposing greater penalties on low IoU matches while maintaining high-quality matching performance, setting a new benchmark in the field of real-time object detection. With the global modeling capability based on Transformer, DEIM can more effectively capture edge information of discrete defects in aluminum alloy wheel images and has stronger anti-interference ability against complex backgrounds. However, despite its advantages in detection accuracy and convergence speed, DEIM still has many deficiencies in the task of aluminum alloy wheel defect detection. The standard HGNetV2 is used as the backbone structure in DEIM, which has a weak response to low-contrast defects and insufficient edge extraction ability, leading to the model being easily disturbed by the background and a decrease in detection capability. In addition, the Efficient Hybrid Encoder in DEIM uses Attention-based Intra-scale Feature Interaction (AIFI) and CNN-based Cross-scale Feature Fusion (CCFF) to handle multi-scale features, aiming to solve the computational efficiency problem in multi-scale feature processing. However, the CCFF module relies on layer-by-layer convolution to achieve cross-scale fusion, and this design cannot effectively aggregate edge detail information when dealing with small targets or targets lacking local details, making it difficult to effectively identify defect edge contours. Moreover, the CCFF does not efficiently implement cross-module feature interaction, causing the model to have difficulty distinguishing background noise when facing defect targets with large scale variations in aluminum alloy wheels, and thus cannot efficiently complete the defect detection task.

Inspired by DEIM, this study proposes a novel detection model for low-contrast discrete defects in aluminum alloy wheel hubs. The effectiveness of the model was verified through ablation experiments and comparative experiments, and a valuable solution for the detection of defects in aluminum alloy wheel hubs under X-ray was provided. The contributions of this study can be summarized as follows:

1. In view of the low contrast between defects and the background and the blurred edges of defects in the original aluminum alloy wheel hub images, data preprocessing methods such as exposure fusion and high-frequency enhancement are adopted to improve the overall contrast of the images. The dataset is expanded through data augmentation methods to enhance the model’s generalization ability. A dataset containing 2318 original high-quality industrial aluminum alloy wheel hub defect images has been constructed, which can serve as a benchmark for further research and validation.
2. To address the problem that standard convolutions in the backbone are difficult to efficiently extract edge information of such defects, the PConv module with a efficient receptive field is introduced to effectively improve the feature extraction capability of the backbone at the bottom layer, thereby improving the model’s feature discrimination capability for discrete defects with extremely low parameter cost.
3. A novel two-stage feature fusion-diffusion pyramid structure named MFDPN has been designed. While ensuring the efficiency of model detection, improve the overall positioning ability of the network for discrete defects. The Structure-Aware Visual State Space (SAVSS) module is introduced to achieve feature interaction and fusion under a richer receptive field. In the MFDPN, Mamba Focus Fusion (MFF) focuses on fusing semantic information from different feature layers to achieve deep feature integration. Diffusion Assist Fusion (DAF) spreads the context-rich features from MFF to different scale branches through a cross-scale feature diffusion mechanism, significantly alleviating the information loss problem caused by scale differences in traditional feature pyramid networks.
4. To address the issue of background information interference during backbone feature extraction, a Channel and Spatial Attention Module (CASAB) is introduced between the backbone and the encoder to enhance the model’s robustness against complex backgrounds.

The structure of this research is as follows: Section 2 details the data acquisition and processing procedures, Section 3 elaborates on the aluminum alloy wheel hub defect detection method proposed in this paper, Section 4 verifies the effectiveness of this method through experiments, and Section 5 summarizes the entire paper.

## 2. Data Acquisition and Processing

**Figure 1:**
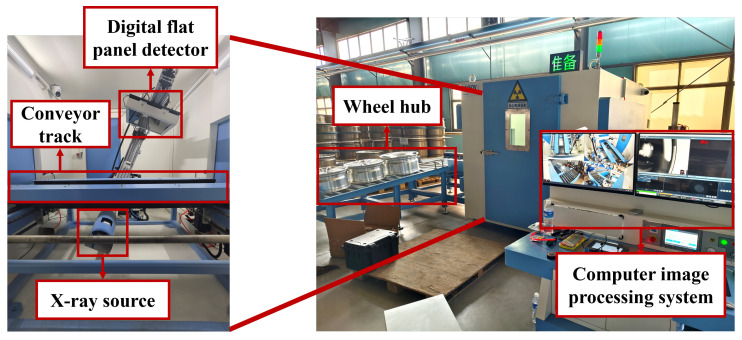
X-ray imaging data acquisition device.

**Figure 2:**
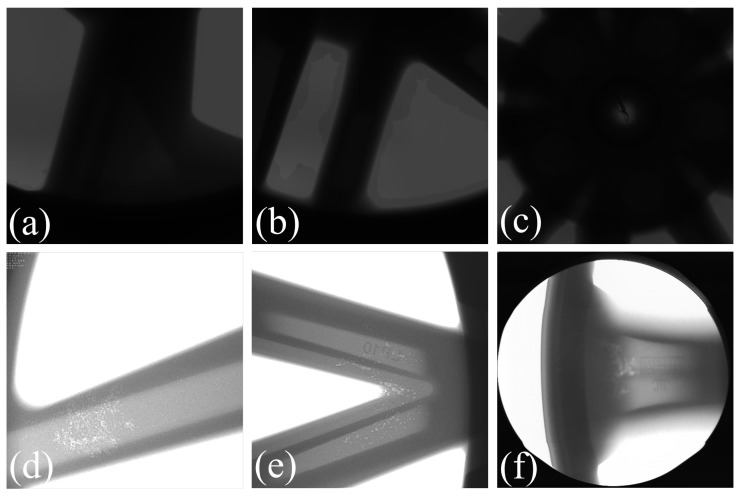
Examples of X-ray images of aluminum alloy wheels. (**a**–**c**) are low-contrast images; (**d**–**f**) are discrete defect images.

Figure 1 shows the X-ray imaging data acquisition device, whose core components include a digital flat panel detector, a conveying track, an X-ray source, the wheel hub to be inspected and a computer image processing system. During operation, the conveying track first sends the wheel hub to be inspected into the X-ray inspection area. The X-ray source emits rays that penetrate the inspected wheel hub. Then, the digital flat panel detector receives the transmitted rays and sends the results to the computer image processing system. Subsequently, the computer processes them into X-ray images. Staff members conduct quality inspections by observing these images. Examples of acquired images are shown in Figure 2.

**Figure 3:**
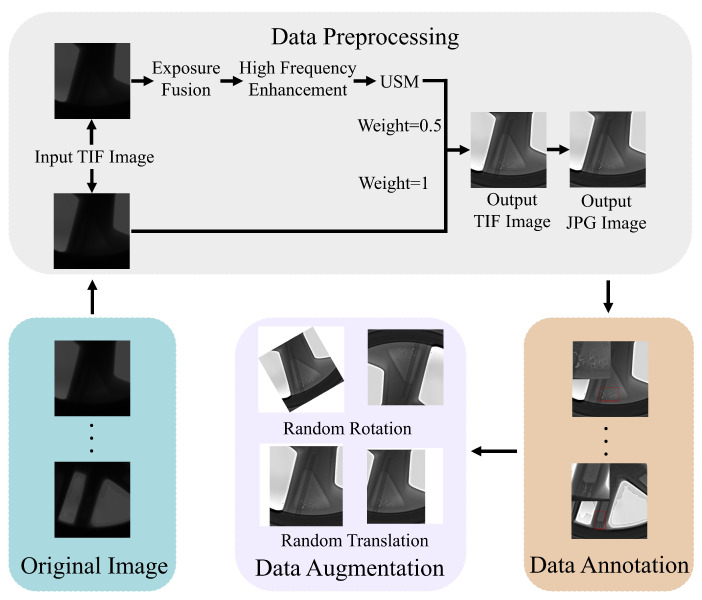
Data processing.

We collected 2318 X-ray images of defective aluminum alloy wheels, with each image measuring 2048 × 2048 pixels. This dataset includes the spoke area, wheel core area, and rim area of the wheel hub, accounting for 37%, 30%, and 33% respectively. As shown in Figure 2, the contrast of the collected raw images is relatively low, and some defects are difficult to label. Therefore, this study first performs data augmentation on the original TIF base images. The specific process is shown in Figure 3, including exposure fusion, high-frequency enhancement, USM sharpening, fusing the processed images with the original images in a certain ratio (set to 0.5:1 here), and finally converting them to JPG format. After processing, the contrast between the background and the defects in the image is enhanced, the edges of the defects become clearer, and the details are more prominent.

**Figure 4:**
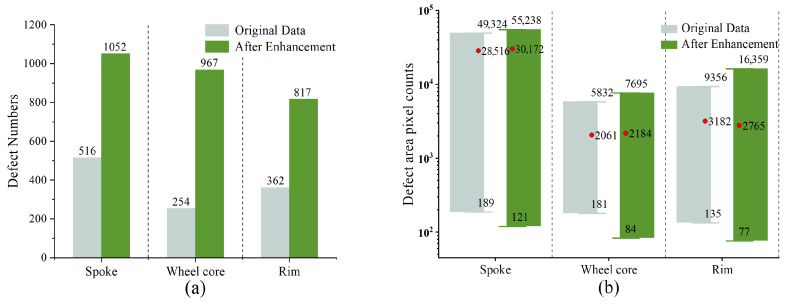
Defect statistics distribution. (**a**) Defect quantity; (**b**) defect area pixel count.

Figure 4 comprehensively presents the defect statistics distribution of each area of the wheel hub in the dataset before and after data augmentation. From Figure 4a, it can be observed that this data augmentation strategy significantly increases the number of defect samples, ensuring the generalization ability of the subsequent model while guaranteeing the model’s optimal detection performance. Figure 4b shows the defect pixel distribution before and after data augmentation. After data augmentation, the range of defect pixels is larger, which is because there are many small sample defects that are difficult to identify by the naked eye and large sample defects with blurred boundaries in the original images. In addition, the average pixel counts of defects on the wheel core and rim are 2184 and 2765 respectively. In contrast, the average pixel count of defects on the spokes is much larger, reaching 30,172. The annotation work of this study was completed by a professional team from the manufacturer using the Labelme annotation software (Version 3.16.2), which took 15 working days. This software stores the classification and coordinate information of each defect in the image in the corresponding json file. Due to the limited original image data, this study used random flipping, translation, and other operations to expand the original dataset to 6954 images, and divided the dataset into training, validation, and test sets in a 7:2:1 ratio.

## 3. Methodology

### 3.1. Our Model

**Figure 5:**
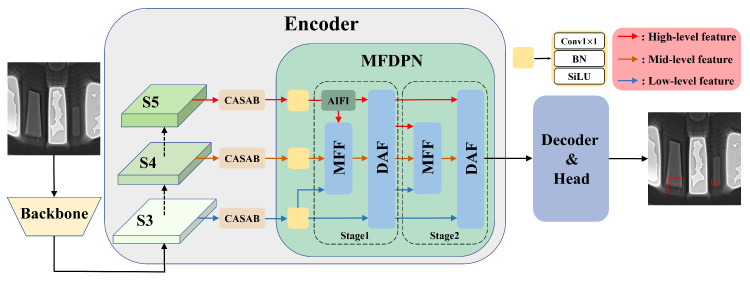
Our model architecture.

As shown in Figure 5, we decompose the model architecture into three main components: backbone, encoder, and decoder. The input image is first processed by the backbone to generate feature maps S3, S4, and S5 at different scales. In the backbone, since standard convolutions are insufficient to fully extract defect features, the PConv module, which has an efficient receptive field, is used. Four different pinwheel-shaped filling methods are used to enhance the backbone’s ability to extract edge features of irregular defects. In the encoder, to address the inability to effectively suppress irrelevant complex background information during backbone feature extraction, the CASAB module is introduced into each feature map. This module, by combining channel attention and spatial attention, guides the model to focus on the core defect region, effectively improving the network’s resistance to interference from complex backgrounds. Then, a 1×1 convolution is applied to unify the number of channels of different feature maps, and they are sent into the MFDPN. MFDPN consists of two stages: In the first stage, the AIFI module processes the high-level feature S5 using a single-scale transformer encoder, aiming to capture the connection between abstract and concrete features and effectively enhance the feature representation ability of subsequent models. The MFF module fuses S3 and the S5 feature map processed by AIFI under the guidance of the S4 feature map to form an initial multi-scale feature representation. The DAF module fuses the features in MFF with S3 and S5 features respectively and diffuses them to the MFF and DAF in the second stage. In the second stage, MFF and DAF conduct secondary deep fusion and diffusion to guide the network to re-integrate the gradient information of each deep layer. Finally, the DAF module of the second stage sends the result to the decoder part, and the decoder predicts the target bounding box and its category based on the feature map.

### 3.2. Pinwheel-Shaped Convolution

**Figure 6:**
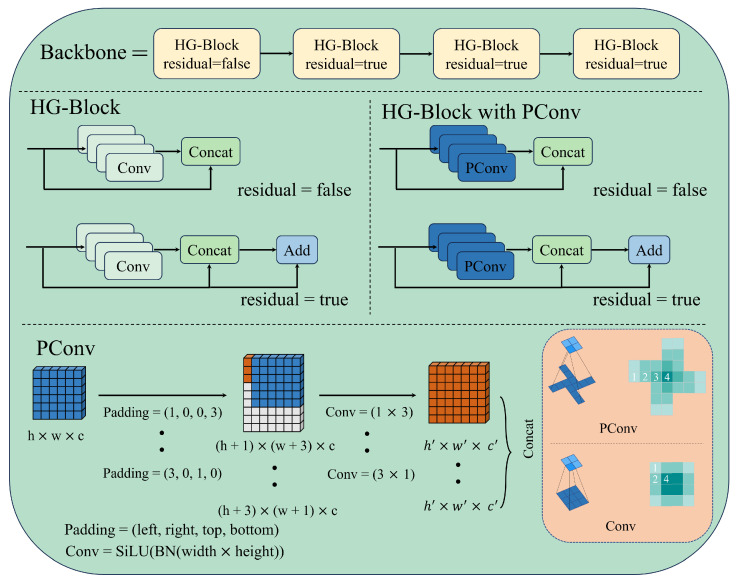
Schematic diagram of pinwheel-shaped convolution module.

As shown in Figure 6, the backbone of DEIM consists of multiple HG-Blocks. Its core is composed of parallel 3×3 convolutional layer residual connections, and its main purpose is to extract features from the input image. However, the feature extraction ability of standard convolution is weak due to its limited by the 3×3 convolution receptive field, which cannot effectively extract defects of random sizes and blurred edges in the dataset, ultimately leading to poor detection performance of the model. Compared with the standard convolution, PConv is a new type of convolutional structure. We replace the standard convolution module in the backbone with the PConv module [21].

PConv convolution employs four parallel padding methods to pad the lower left, upper right, lower right, and upper left of the feature map respectively, with the aim of creating horizontal and vertical convolution kernels for different regions of the image. The convolution kernels are radially diffused and are divided into two types: 1×3 and 3×1. To enhance training stability, normalization and SiLU activation function processing are uniformly applied after convolution. In the figure, *h*, *w*, and *c* respectively represent the length, width, and number of channels of the input feature map. The specific calculation process is as follows:

(1) $$X_{1}\left(h^{\prime },w^{\prime },c^{\prime }\right)=\mathrm{SiLU}\left(\mathrm{BN}\left(X_{P(1,0,0,3)}^{\left(h,w,c\right)}{\mathrm{Conv}}_{c^{\prime }}\left(1\times 3\right)\right)\right),$$

(2) $$X_{2}\left(h^{\prime },w^{\prime },c^{\prime }\right)=\mathrm{SiLU}\left(\mathrm{BN}\left(X_{P(0,3,0,1)}^{\left(h,w,c\right)}{\mathrm{Conv}}_{c^{\prime }}\left(3\times 1\right)\right)\right),$$

(3) $$X_{3}\left(h^{\prime },w^{\prime },c^{\prime }\right)=\mathrm{SiLU}\left(\mathrm{BN}\left(X_{P(0,1,3,0)}^{\left(h,w,c\right)}{\mathrm{Conv}}_{c^{\prime }}\left(1\times 3\right)\right)\right),$$

(4) $$X_{4}\left(h^{\prime },w^{\prime },c^{\prime }\right)=\mathrm{SiLU}\left(\mathrm{BN}\left(X_{P(3,0,1,0)}^{\left(h,w,c\right)}{\mathrm{Conv}}_{c^{\prime }}\left(3\times 1\right)\right)\right)$$

Here, $X_{i}\left(h^{\prime },w^{\prime },c^{\prime }\right)$ represents the output feature map obtained by the *i*-th padding method, (h,w,c) represents the input feature map, and P(1,0,0,3) respectively indicates the number of padding pixels on the left, right, top, and bottom. ${Conv}_{c^{\prime }}\left(1\times 3\right)$ represents a 1×3 convolution kernel with output channels of $c^{\prime }$. Finally, the four output feature maps are concatenated through Concat to obtain the final result $X^{\prime }$:

(5) $$X^{\prime }\left(h^{\prime },w^{\prime },4c^{\prime }\right)=\mathrm{Concat}\left(X_{1}\left(h^{\prime },w^{\prime },c^{\prime }\right),\ldots ,X_{4}\left(h^{\prime },w^{\prime },c^{\prime }\right)\right)$$

As shown in the lower right of Figure 6, when the convolution kernel size is 3, the receptive field of PConv is 25, which is a 177% increase compared to that of a regular convolution. In the backbone of DEIM (where the number of output channels $C_{2}$ is four times the number of input channels $C_{1}$), the parameter count of the standard convolution is $36C_{1}^{2}$, while that of PConv is $72C_{1}^{2}$. With a 111% increase in parameters, the receptive field is enhanced by 178%. This indicates that PConv significantly improves the efficient receptive field expansion of the model at a very low parameter cost. In the dataset of this article, most defects are of diffused and irregular shapes, and the receptive field design of PConv can effectively learn the edge features of such defects. Furthermore, from the number of convolution times in the receptive fields, it can be found that PConv pays more attention to edge computing than standard convolution. The number of receptive fields with one convolution time in PConv is 8, while in standard convolution it is 4; the number of receptive fields with two convolution times in PConv is 12, while in standard convolution it is 8. This indicates that PConv pays more attention to defect edge information than standard convolution.

### 3.3. Mamba Docus Diffusion Pyramid Network

The backbone network contains different network feature layers from shallow to deep. Accurate localization and classification not only rely on the detailed edge information provided by the shallow layers but also require the deep layers to capture the overall information. We propose a two-stage Mamba Focus Diffusion Pyramid Network (MFDPN) structure, where each stage includes Mamba Focus Fusion (MFF) and Diffusion Assist Fusion (DAF) modules, aiming to achieve efficient integration of multi-scale features across modules and enhance the model’s ability to identify defects with blurred edges and random sizes.

#### 3.3.1. Mamba Focus Fusion

**Figure 7:**
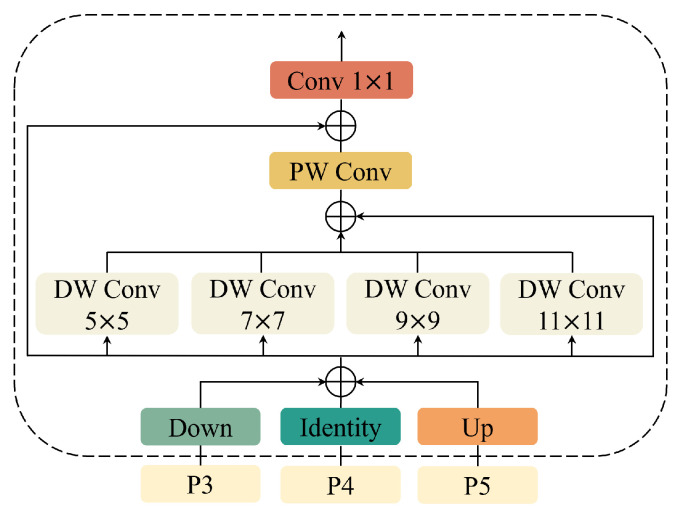
Architecture of DW_Conv module.

To comprehensively focus on the cross-level and cross-position information of the backbone network, a commonly adopted approach is to use a parallel depthwise separable convolution structure such as (5×5, 7×7, 9×9, 11×11). As shown in Figure 7, although this static structure can enhance the network’s ability to capture spatial information, when multiple convolution blocks are processed in parallel, it may lead to computational redundancy and reduced efficiency on one hand, and on the other hand, there is no interaction among the channels of different branches, which significantly reduces the network’s feature aggregation capability, making it difficult for the network to identify the diffusive defects in the wheel hub. Therefore, in this section, a MFF module based on Mamba is proposed, which not only improves the utilization rate of network parameters but also enhances the network’s dynamic sequence modeling ability and dynamic global information aggregation ability.

**Figure 8:**
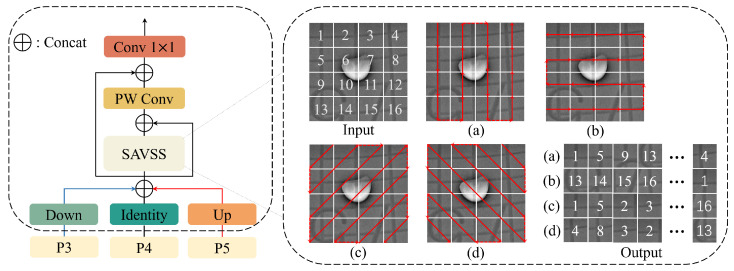
MFF module architecture. (**a**) represents column scanning; (**b**) represents row scanning; (**c**) represents diagonal snake scanning and (**d**) represents anti-diagonal snake scanning.

As shown in Figure 8, we first unify the resolutions of the feature maps P3, P4, and P5 in the backbone to the size of P4 and concatenate them together. Then, they pass through a residual block composed of SAVSS modules [22]. In this module, we abandon the multi-convolution parallel structure and adopt the visual Mamba approach to extract features. Specifically, we use two parallel snake-shaped scanning strategies (a and b) and two diagonal snake-shaped scanning strategies (c and d) for feature extraction. This feature extraction method breaks away from the limitations of the traditional convolutional receptive field and remotely establishes multi-directional adjacency relationships of defects through a dynamic global receptive field, thereby enhancing the network’s ability to recognize irregular defects. This design structure, while controlling the number of model parameters, has a stronger parameter utilization rate than the multi-convolution parallel structure. The residual structure not only accelerates the convergence speed of the model but also enhances the model’s expressive power, enabling deep networks to efficiently represent abstract features. Subsequently, PW Conv further fuses the features processed by the SAVSS module with the original features, maintaining the number of channels before and after. Finally, it is sent to a 1×1 convolution for dimensionality reduction and passed into the DAF module.

Let the size of the P4 feature map be H×W×C, and $P_{c}$ be the sum of the input feature maps P3, P4, and P5, with a size of H×W×3C. *P* represents the output result. The symbol $C_{1}\left(\cdot \right)$ indicates a 1×1 convolution, PW(·) represents a pointwise convolution, S(·) represents the SAVSS module, and ⊕ indicates concatenation. The output result after MFF processing is:

(6) $$P=C_{1}\left(P_{c}⊕\left(PW\left(P_{c}⊕S\left(P_{c}\right)\right)\right)\right)$$

#### 3.3.2. Diffusion Assist Fusion

**Figure 9:**
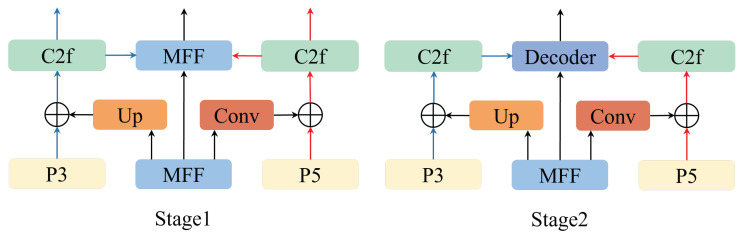
DAF module architecture.

To further enhance the model’s ability to aggregate features for large-area irregular defects, we added a diffusion-assisted fusion (DAF) model to MFDPN. Specifically, Figure 9 shows the two-stage DAF module. In the first stage, DAF receives the depth-focused fusion information processed by MFF in the first stage and diffuses it to the P3 and P5 layers after up-sampling and down-sampling respectively. This diffusion mechanism not only effectively prevents the occurrence of model overfitting, but also ensures that each feature map at different scales contains both the local detail texture information and the global semantic information of the defect through the fusion of high-resolution and low-resolution images. Then, the features are initially integrated through the deep feature enhancement module (C2f) and sent respectively to the MFF and DAF in the second stage. In addition, we also map the original features of MFF identically to the MFF in the second stage, which can enrich the depth features of MFF in the second stage. In the second stage, DAF again assists in integrating the depth information in MFF in the same way and sends the final result to the Transformer Decoder, providing a richer feature representation for the subsequent target detection, localization and classification.

Let $P_{3}^{\prime }$, $P_{M}^{\prime }$ and $P_{5}^{\prime }$ be the three outputs from the P3 layer to the P5 layer in the first stage of DAF. U(·) represents up-sampling convolution, D(·) represents down-sampling convolution, and C(·) represents the C2f module. Then, the three outputs of the first stage are respectively:

(7) $$P_{3}^{\prime }=C\left(P_{3}⊕U\left(P\right)\right),$$

(8) $$P_{M}^{\prime }=P,$$

(9) $$P_{5}^{\prime }=C\left(P_{5}⊕D\left(P\right)\right)$$

Similarly, let $P_{3}^{\prime \prime }$, $P_{M}^{\prime \prime }$ and $P_{5}^{\prime \prime }$ be the three outputs of the second stage of DAF, and $P^{\prime }$ be the output of the second stage of MFF. Then, the outputs of the second stage are respectively:

(10) $$P_{3}^{\prime \prime }=C\left(P_{3}^{\prime }⊕U\left(P^{\prime }\right)\right),$$

(11) $$P_{M}^{\prime \prime }=P^{\prime },$$

(12) $$P_{5}^{\prime \prime }=C\left(P_{5}^{\prime }⊕D\left(P^{\prime }\right)\right)$$

### 3.4. Channel and Spatial Attention Block

The backbone extracts defect features from complex images through convolution. As the feature extraction capability of the backbone network improves, it also contains a large number of redundant background features. This redundant information can mislead the MFDPN pyramid network to learn unnecessary background features, reducing the detection efficiency of the model. To address the above problems, we introduce a channel and spatial attention block [23] between the backbone and MFDPN to improve the model’s ability to resist interference from complex backgrounds and improve detection accuracy.

**Figure 10:**
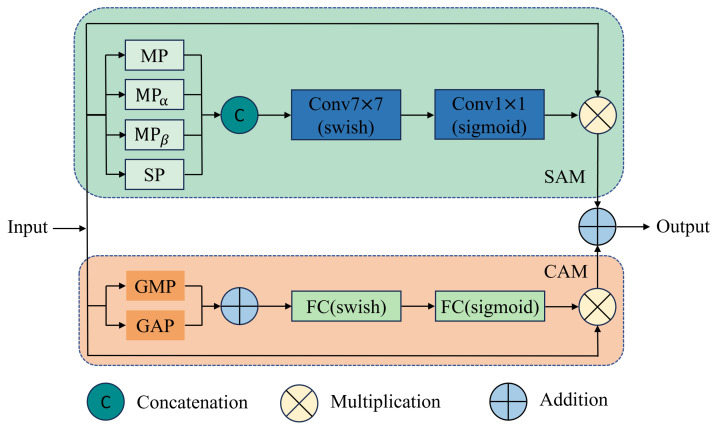
Structure of CASAB.

As shown in Figure 10, this module selectively strengthens the most informative key features in the channel and spatial dimensions by integrating channel and spatial attention mechanisms, enabling the model to efficiently focus on defect features and weaken background features. In CAM, Global Average Pooling (GAP) is responsible for capturing overall image information, while Global Max Pooling (GMP) extracts defect information from the image. The two pieces of information are then added together, followed by two fully connected layers containing swish functions for non-linear activation and gradient smoothing. Finally, attention weights are generated using a sigmoid activation function and multiplied by the original input feature *x*. The formula for calculating CAM is:

(13) $$CAM\left(x\right)=x\cdot FC\mathrm{sigmoid}\left(\left(FC\mathrm{swish}\left(GAP(x)+GMP(x)\right)\right)\right)$$

In SAM, the input features are processed by mean pooling (MP), max pooling ${(\mathrm{MP}}_{\alpha })$, min pooling ${(\mathrm{MP}}_{\beta })$, and summation pooling (SP) to capture the existence, significance, weakest response, and overall strength of discrete defects in spatial features. Subsequently, 7×7 convolution is applied to capture broader defect information, which is then processed by the swish and sigmoid activation functions before being finally multiplied by the original input feature *x*. The formula for calculating SAM is:

(14) $$SAM\left(x\right)=x\cdot \mathrm{sigmoid}\left({\mathrm{Conv}}_{1\times 1}\left(\mathrm{swish}\left({\mathrm{Conv}}_{7\times 7}\left(C\left(\mathrm{MP}\left(x\right),{\mathrm{MP}}_{\alpha }\left(x\right),{\mathrm{MP}}_{\beta }\left(x\right),\mathrm{SM}\left(x\right)\right)\right)\right)\right)\right)$$

where *C* represents Concatention. The final output of CASAB is:

(15) Output=CAM(x)+SAM(x)

## 4. Experiments

### 4.1. Experimental Environment and Training Strategy

To ensure the reliability of the experimental results, all experiments in this study were conducted under a unified hardware and software environment. The experimental platform was based on the Linux operating system and equipped with an NVIDIA RTX4090 graphics card (NVIDIA, Santa Clara, CA, USA). The specific configuration information of the experimental environment is shown in Table 1. The experimental model in this study does not rely on large-scale datasets such as ImageNet or pre-trained weights. To better compare DEIM with the model proposed in this paper, we basically followed the training strategy of DEIM-S. The specific training parameter information is shown in Table 2.

### 4.2. Objective Evaluation Indicators

This work employs mean Average Precision (mAP), Recall, FPS, and Parameters as evaluation metrics, which have been widely adopted for object detection tasks. Additionally, we define mAP metrics for small objects (area $\leq 32^{2}$ pixels), medium objects ($32^{2}\leq$ area $\leq 64^{2}$ pixels), and large objects (area $\geq 64^{2}$ pixels) based on the defect area of the target. Furthermore, we employ computational complexity measured by Floating Point Operations (FLOPs) as an indicator of model detection speed. The calculation formulas for each evaluation metric are as follows:

(16) $$mAP=\frac{\sum_{i=1}^{n}AP_{i}}{n},$$

(17) $$Recall=\frac{TP}{TP+FN},$$

(18) $$FPS=\frac{1}{t}$$

Here, $AP_{i}$ denotes the accuracy rate for the *i*-th category, *n* represents the total number of sample categories, TP indicates the number of positive samples correctly detected by the model, FP signifies the number of negative samples falsely predicted as positive by the model, FN refers to the number of positive samples incorrectly classified as negative by the model, and *t* denotes the time required to process an image, measured in seconds.

### 4.3. Ablation Experiments

We first conducted an ablation study on the data augmentation part of the data preprocessing section, with the DEIM-S model being the default for training. As shown in Table 3, when training with the original images, the Recall accuracy was the lowest. This is because the contrast between the background and defects in the original images was low, which led to poor performance of the network in distinguishing defects from the background. When training with images that have undergone exposure fusion, high-frequency enhancement, and USM sharpening, the recall and accuracy rates have improved by 1.2% and 0.9% respectively. In addition, we also conducted experimental analysis on the proportional fusion of the original image and the enhanced image (α = 0.5 indicates that the original image and the processed image are fused at a ratio of 1:0.5). It can be found that when α = 0.5, the effect is optimal, indicating that the network’s detection ability for low-contrast aluminum alloy wheel hub defect images reaches its best at this time.

As shown in Table 4, we conducted ablation experiments on the baseline model DEIM-S. First, we adopted the PConv module in the backbone network, improving network performance by 3.3% with only a 0.3 M increase in parameters. Notably, the PConv module improved the accuracy for small, medium, and large targets by 3.3%, 3.1%, and 2.6%, respectively, demonstrating that PConv improves the model’s defect detection performance with extremely low parameter costs. Next, we adopted the MFDPN module in the encoder, further improving performance by 3.4% with only a 2.4 M parameter cost. Specifically, the recall rate improved by 1.2%, and the accuracy for small, medium, and large targets improved by 3.4%, 3.4%, and 4%, respectively, proving that MFDPN significantly enriches the model’s multi-scale representation capabilities and significantly improves the model’s detection accuracy while maintaining low computational cost. Finally, the inclusion of the CASAB module resulted in the highest detection accuracy. With almost no increase in parameters, the detection accuracy improved by 0.5%, reaching 91.1%. This indicates that the CASAB module can effectively enhance the model’s anti-interference ability and highlight the defect location region.

Furthermore, we conducted an ablation study on the two-stage MFDPN, with the model defaulting to the Efficient Hybrid Encoder structure. As shown in Table 5, we first adopted the one-stage MFDPN structure in the encoder. Despite a reduction of 0.5 M in parameters, the network performance still improved by 2.2%, with accuracy rates for small, medium, and large targets increasing by 2.8%, 1.8%, and 2.1% respectively. This demonstrates that MFDPN effectively enhances the defect detection capability of the model while maintaining its timeliness. Then, we applied a two-stage MFDPN structure in the Encoder. Thanks to its efficient feature fusion design, the performance improves by another 4.1% at the cost of only 3 M additional parameters. The recall rate increases by 1.9%, and the accuracy rates for small, medium, and large targets increase by 3.9%, 4.5%, and 4% respectively. Moreover, the FPS remains above 40. This proves that the dual-stage MFDPN module not only improves the defect detection accuracy of the model but also maintains a very low parameter cost and computational load, verifying the effectiveness of its design.

### 4.4. Comparative Experiments

To verify the effectiveness of the SAVSS module in MFF, we conducted a comparative study on the two-stage MFF module. As shown in Table 6, we compared the two-stage DW parallel convolution module and the SAVSS module, where the structure of the DW convolution module is illustrated in Figure 7. It can be seen from Table 6 that the two-stage DW parallel convolution module performs worst, especially in detecting small targets, indicating the limitations and low adaptability of standard convolution in the hub defect detection task. The first-stage DW parallel convolution module and the second-stage SAVSS module perform slightly better than the first-stage SAVSS module and the second-stage DW parallel convolution module. This might be because low-level features usually contain a large amount of detail noise and texture, which is not conducive to Mamba processing a large amount of redundant raw detail information. The two-stage SAVSS module has achieved the optimal effect, with a 5.2% improvement over the two-stage DW parallel convolution module. Additionally, the recall rate has increased by 3.7%, and the accuracy rates for small, medium, and large targets have respectively improved by 5.4%, 4.6%, and 3.2%. This indicates that the defect detection effect of the SAVSS module is significantly stronger than that of the DW parallel convolution structure, and the FPS can still be maintained at around 40, meeting the actual industrial requirements.

**Figure 11:**
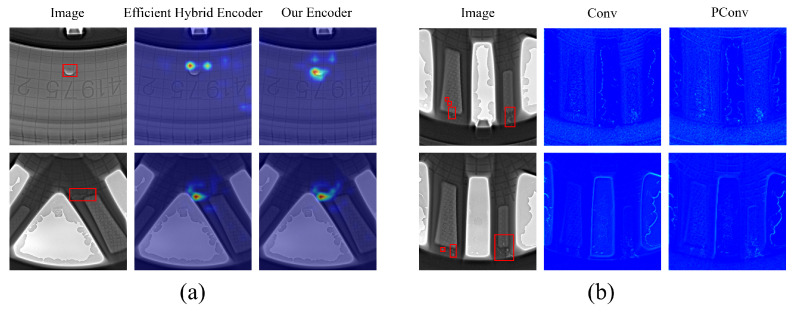
Visual comparison results of heatmap and feature map. (**a**) Comparison of encoder defect localization; (**b**) Comparison of mainframe defect feature extraction.

To verify the effectiveness of the proposed model, we added a heatmap to the encoder and compared it with DEIM’s Efficient Hybrid Encoder. As shown in Figure 11a, the red rectangle in the figure represents the defect region. It can be seen that the proposed model is significantly stronger than the Efficient Hybrid Encoder in terms of the defect-focused region, which demonstrates the effectiveness and superiority of the proposed model in defect localization. In addition, we added a feature map to the backbone and compared it with standard convolution. As shown in Figure 11b, the red rectangle represents the defect region. The backbone feature extraction capability of PConv is significantly stronger than that of standard convolution.

Table 7 presents the comparison results of our proposed model with other state-of-the-art real-time object detectors. We first compared it with the baseline model (DEIM-S), and our model achieved improvements of 5%, 7.2%, 7.1%, 7.2%, and 7.1% respectively in the metrics. Compared to other YOLO series, the model in this paper demonstrates a better balance between accuracy and computational cost. For instance, compared with Gold-YOLO-S, the model in this paper reduces the number of parameters by 39% while still maintaining a performance improvement of 14.5%, showcasing an outstanding parameter utilization rate. Furthermore, we compared our model with transformer-based real-time object detectors, and thanks to our ingenious model design, our model demonstrated superior performance in all cases, confirming that our model can achieve high-performance detection with low resource consumption. The above experimental results show that the model proposed in this paper can significantly improve the detection accuracy of defects in aluminum alloy wheels while maintaining a relatively fast detection speed, meeting the requirements of actual industrial production environments.

**Figure 12:**
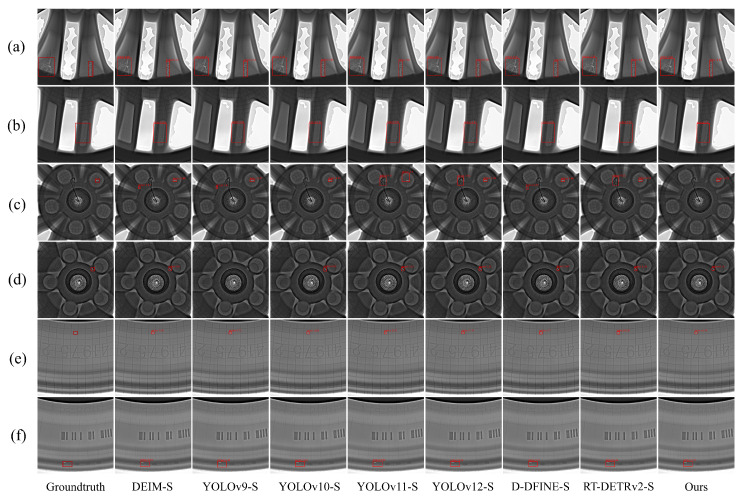
Detection results of the real-time target detector at different wheel hub positions. (**a**,**b**) represent the spoke area; (**c**,**d**) represent the wheel core area; (**e**,**f**) represent the rim area.

Figure 12 shows the detection results of our proposed model and other real-time target detectors on different parts of the wheel hub, where (a) and (b) represent the spoke region, (c) and (d) represent the wheel core region, and (e) and (f) represent the rim region. The figure shows that for large-area defects in the spokes, our proposed model outperforms other models, which is attributed to the powerful feature integration and feature diffusion capabilities of MFDPN. For defects in the wheel core, the high background complexity makes defect identification difficult, leading to false positives and false negatives in other models. In contrast, our proposed model does not exhibit these issues, thanks to the excellent anti-interference capabilities of the CASAB module. For small defects in the rim, our proposed model still achieves better detection results, indicating that PConv convolution can significantly improve the model’s feature sensing ability for defect edges. These visualization analyses effectively verify that the proposed model has superior detection capabilities for defects in aluminum alloy wheel hubs.

**Figure 13:**
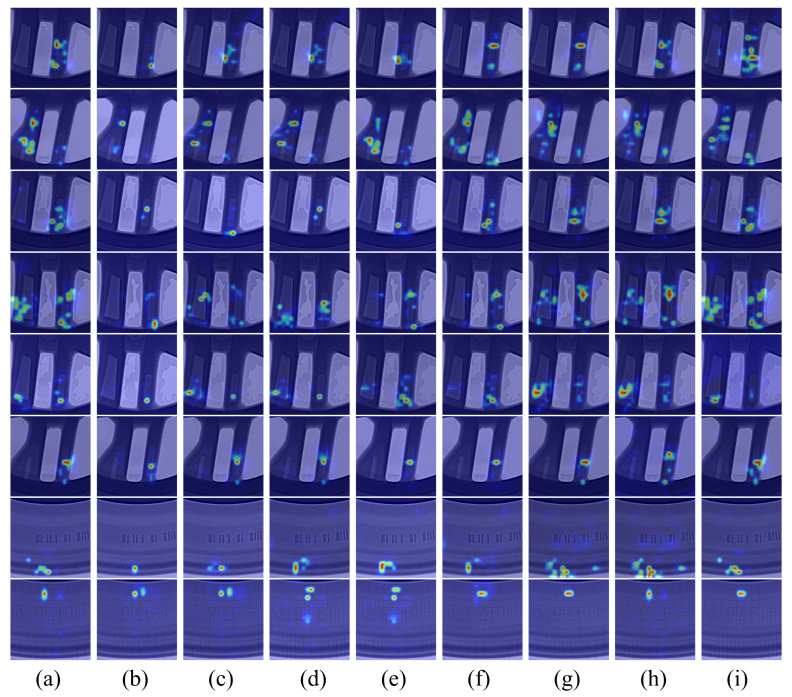
Comparison of model heatmaps. (**a**–**i**) represent DEIM-S, YOLOv8-S, YOLOv9-S, YOLOv10-S, YOLOv11-S, YOLOv12-S, D-DFINE-S, RT-DETRv2-S, and our model respectively.

To visually demonstrate the detection performance of our proposed model, we compared and analyzed the heatmaps of different real-time target detectors, as shown in Figure 13. Compared to the YOLO series models, our proposed model exhibits a higher response intensity to defect regions and a more coherent attention distribution, indicating a stronger ability to identify target defects. Compared to the Transformer series models, our proposed model shows a more concentrated attention distribution to defect regions and is less affected by complex backgrounds, indicating a higher focus intensity on defect regions and stronger anti-interference capabilities. In conclusion, our proposed model achieves optimal performance on the aluminum alloy wheel hub defect dataset.

## 5. Conclusions

This study addresses the poor detection performance of aluminum alloy wheel hubs due to low contrast between defects and background and the discrete distribution of defect shapes in images. A novel defect detection model for aluminum alloy wheels is proposed, with the following contributions: (1) Image preprocessing methods such as exposure fusion and high-frequency enhancement are employed to improve the contrast between defects and the background in the aluminum alloy wheel dataset. (2) The PConv module is used in the backbone network to significantly enhance the model’s feature extraction capabilities for discrete defect edges with extremely low parameter cost. (3) To achieve efficient integration of multi-scale features across modules and improve the model’s feature integration capabilities for discrete defects, an innovative Mamba-based MFDPN structure is proposed. This structure promotes extensive interaction and diffusion of multi-scale information across different feature layers in the encoder, effectively mitigating information loss due to scale differences in traditional pyramid networks and significantly improving the feature aggregation capabilities of the network’s fusion layers. (4) The CASAB module is introduced to improve the model’s resistance to interference from complex backgrounds and enhance detection accuracy. Finally, we systematically integrate the above structures to construct a novel target detection model for aluminum alloy wheel hub defect datasets. Extensive experimental analysis shows that the model proposed in this paper outperforms the current state-of-the-art real-time object detectors in several key metrics. Specifically, mAP50 is improved by 7.2% compared to the baseline model, and the detection accuracy for small, medium, and large objects is improved by 7.1%, 7.2%, and 7.1%, respectively. Recall is improved by 5%, and the FPS is 39. This meets the detection requirements of actual aluminum alloy wheel factories.

In engineering deployments, the model proposed in this paper can be integrated into industrial defect detection equipment terminals. By outputting high-quality defect detection data, it can effectively improve product quality, extend the service life of wheel hub structures, and provide strong support for intelligent manufacturing and critical infrastructure monitoring. While the model presented in this paper demonstrates significant advantages in aluminum alloy wheel hub defect detection, it still has certain limitations: First, the framework only supports two-dimensional ray image processing and has not yet effectively learned three-dimensional defect data features, making it difficult to accurately capture the depth distribution and spatial morphology of defects inside the wheel hub; second, the current implementation has not been extended to multimodal processing, lacking cross-modal analysis with technologies such as infrared thermal imaging and ultrasonic detection, and failing to integrate the advantages of different detection technologies to achieve complementary and verified defect information. In future research work, a three-dimensional convolutional network will be introduced to expand the existing model’s three-dimensional feature learning capability, in order to accurately restore the spatial position, depth and volume information of defects. We will also focus on providing more comprehensive data support for the strength assessment of wheel hub structures; integrating X-ray, infrared thermal imaging and ultrasonic testing data; designing a cross-modal feature fusion module to improve the detection accuracy of complex defects; and further expanding the application value of the model in intelligent manufacturing scenarios.

## Figures and Tables

**Table 1:** Experimental environment and parameter settings.

Configuration Name	Configuration Parameters
Operating System	Ubuntu 22.04 Linux
GPU	NVIDIA RTX4090 (24 G)
CPU	i7-13700F CPU
Memory	128 G
Software	Pycharm2023
CUDA	11.8
Python	3.8
PyTorch	2.2.0

**Table 2:** Key parameter settings.

Parameter	Value
Optimizer	AdamW
Batchsize	16
Image Size	640 × 640
Initial learning rate	0.0002
Final learning rate	0.0001
Epoch	120

**Table 3:** Ablation experiment study on data augmentation.

α	EF	HFE	USM	Recall	AP_50_
				88.3	83.0
0.5	✓			88.0	82.7
0.5	✓	✓		89.1	83.2
0.5	✓	✓	✓	89.5	83.9
0.4	✓	✓	✓	88.9	83.4
0.6	✓	✓	✓	89.2	83.6
0.8	✓	✓	✓	88.5	83.1

**Table 4:** Ablation experiment study.

Data Aug	PConv	MFDPN	CASAB	Param	FPS	Recall	AP_50_	AP_*S*_	AP_*M*_	AP_*L*_
				10.2 M	49	88.3	83.0	36.2	72.1	83.6
✓				10.2 M	49	89.5	83.9	37.0	73.2	84.2
✓	✓			10.5 M	48	91.9	87.2	40.3	76.3	86.8
✓		✓		12.7 M	41	93.3	90.2	43.7	79.5	90.3
✓			✓	10.3 M	48	90.2	86.8	39.6	75.1	85.6
✓	✓	✓		12.9 M	39	93.1	90.6	43.7	79.7	90.8
✓	✓		✓	10.7 M	47	92.1	87.5	39.9	76.7	87.4
✓	✓	✓	✓	13.1 M	39	94.5	91.1	44.1	80.4	91.3

Data Aug represents data augmentation when α = 0.5.

**Table 5:** Ablation experiment study of MFDPN.

S-1	S-2	Param	FPS	Recall	AP_50_	AP_*S*_	AP_*M*_	AP_*L*_
		10.2 M	49	89.5	83.9	37.0	73.2	84.2
✓		9.7 M	52	91.4	86.1	39.8	75.0	86.3
✓	✓	12.7 M	41	93.3	90.2	43.7	79.5	90.3

S-1 indicates the first stage, and S-2 indicates the second stage.

**Table 6:** Comparative experimental research on MFF module.

S-1		S-2		Param	FPS	Recall	AP_50_	AP_*S*_	AP_*M*_	AP_*L*_
DW	MFF	DW	MFF							
✓		✓		12.0 M	44	90.8	85.9	38.7	75.8	88.1
✓			✓	12.5 M	42	93.1	87.4	40.2	78.4	90.1
	✓	✓		12.5 M	42	92.6	86.0	39.1	77.9	89.3
	✓		✓	13.1 M	39	94.5	91.1	44.1	80.4	91.3

S-1 indicates the first stage, and S-2 indicates the second stage.

**Table 7:** Comparative experimental study of the proposed model and different advanced real-time object detectors.

Model	Param	FLOPs	Recall	AP_50_	AP_*S*_	AP_*M*_	AP_*L*_
DEIM-S [19]	10.2 M	24.8 G	89.5	83.9	37.0	73.2	84.2
Gold-YOLO-S [24]	21.5 M	46.0 G	83.1	76.6	33.1	65.2	77.3
YOLOv8-S	11.2 M	28.6 G	82.4	76.1	32.9	64.6	77.1
YOLOv9-S [25]	7.2 M	26.7 G	82.7	77.5	33.3	66.8	78.1
YOLOv10-S [26]	7.2 M	21.6 G	83.2	78.2	34.1	67.5	79.4
YOLO11-S [27]	9.4 M	21.5 G	84.7	79.9	35.3	70.2	80.9
YOLOv12-S [28]	9.3 M	21.4 G	84.0	78.5	34.9	68.1	80.1
D-DFINE-S [29]	10.2 M	25 G	88.9	81.2	36.2	72.3	82.8
RT-DETRv2-S [30]	20.0 M	60 G	88.5	80.2	35.8	71.4	82.4
Faster-RCNN-R101 [31]	60.7 M	255 G	80.6	69.4	30.2	57.3	70.2
Swin-T(Cascade-Mask-RCNN) [32,33]	86.0 M	745 G	94.8	92.7	-	-	-
Ours	13.1 M	29.4 G	94.5	91.1	44.1	80.4	91.3

## Data Availability

Data are not publicly available but can be obtained by contacting the corresponding author if necessary.
